# Embedded Immunodetection System for Fecal Occult Blood

**DOI:** 10.3390/bios11040106

**Published:** 2021-04-03

**Authors:** Kai-Wen Lin, Yu-Chi Chang

**Affiliations:** Department of Engineering Science, National Cheng Kung University, Tainan 701, Taiwan; n98081050@gs.ncku.edu.tw

**Keywords:** rapid test, immunodetection system, fecal occult blood

## Abstract

In this paper, a rapid test system with high sensitivity, linearity, and stability is presented for fecal occult blood (FOB) detection. The coloration results of the immune response are used as the basis for the determination of the detection target in combination with an immunochromatographic strip. The rapid test system can be used to detect and calculate the concentration of the sample, so detection of the immune coloration response is more accurate in a quantitative analysis. The system is composed of both hardware and software. The programs used for the analysis and programmed by Python include the main program, polarization calibration, QR Code decoding, Bluetooth transmission, and image processing. After verification of each part of the system, it was found that the rapid test system successfully detects from 0 ng/mL to 400 ng/mL of FOB with coefficients of variation (CV) below 3.7% and 1000 ng/mL with a CV only at 7.41%.

## 1. Introduction

A fecal occult blood (FOB) test is clinically suitable for detecting bleeding in patients with gastrointestinal diseases, such as peptic ulcer, intestinal inflammation, colorectal polyps, and colorectal cancer. According to studies in the literature, an FOB test can reduce the incidence or mortality of colorectal cancer [1,2]. The most commonly used screening method is to test the FOB as a screening item [3]. Precancerous adenomas are likely to evolve into adenocarcinoma, where in most cases, they are adenocarcinoma. The process by which rectal cancer forms is typically the accumulation and evolution of normal mucosa through a series of carcinogenic and tumor suppressor gene changes. Therefore, the purpose of screening is to detect early-stage cancer and treat it.

**Figure 1:**
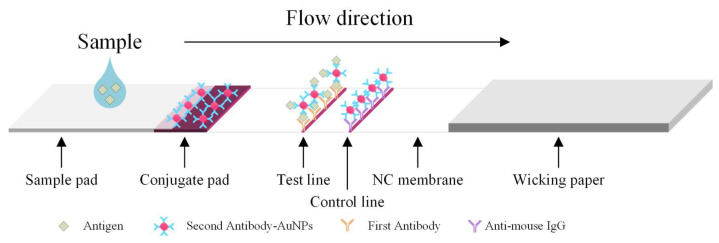
Principle of the lateral flow test.

The major disadvantages of ELISA include the fact that it is labor-intensive; there is a high possibility of false positive/negative results; there is insufficient blocking of immobilized antigens, which results in false results; and refrigerated transport and storage are required for the antibodies [4]. Colloidal gold immunochromatography is a commonly used immunoblotting technique in clinical practice that is also used for rapid detection. Even though an immunoassay based on lateral flow operates on the same principle as the ELISA, it has many advantages, including the fact that it is inexpensive, delivers fast results (within 5 to 15 min), and does not require processing in a laboratory. Therefore, it is superior to the conventional ELISA and has relatively good sensitivity reproducibility [5,6]. Figure 1 shows the major structures and working principle of the lateral flow rapid test. The liquid sample containing blood, if any, flows from the sample pad to wicking paper. The hemoglobin binds the second antibody-AuGPs (gold nanoparticles) conjugates. They are then captured by the first antibody immobilized on the test line (T line). The T line exhibits a color change due to the aggregated AuNPs.

Prior to the development of image analyses for rapid tests, the analytical method relied only on visual qualitative or semi-quantitative analyses, in which the standard colorimetric card and the color of the T line on the rapid test are compared [7,8]. These methods limit the scope of clinical applications because the basis for judging the results is direct observation using the naked eye, which may lead to subjective misjudgment.

A colorimetric card is used as an auxiliary standard for color judgment. However, the human eye varies in terms of color resolution and the limits that can be judged, and color is also affected by light and the external environment, so it is difficult to accurately quantify FOB concentrations when there is only a slight change in color. It is often necessary to use a standard colorimetric card to compare the test results when making judgements at low concentrations; however, when the FOB concentration is low, the human eye is often unable to clearly distinguish the difference between two similar concentrations.

In 2002, Zhang et al. developed a color image detector based on CCD and DSP hardware in order to perform a three-color data calculation and rebuild color patterns [9]. In 2005, Zhou et al. also developed automatic white balance for digital cameras [10]. However, these technologies had not been applied in the field of biomedicine at that time. Zheng et al. applied image processing to immunodetection in 2010. They chose the green component as the representative of the three primary colors, red, green, and blue (RGB), took the green component as the basis for density judgment, and chose the strong edge part as the judgment criterion [11]. In 2011, Jiang and Du suggested that taking pictures and counting the intensity ratio of the pixels (i.e., grayscale measurement) was sufficient as a basis for judgment [12], so they also wanted to use the fuzzy C-means algorithm based on a computer image analysis system.

Therefore, to meet current clinical requirements, quantitative detection of targeted specimens has become necessary, and the development of rapid tests and image detection equipment has thus gained broad interest. Image-based immunochromatographic test strip readers have already been demonstrated by a few researchers [13,14,15,16].

Researchers have also used more reliable or quantitative methods as a benchmark for judgments. For example, the use of a colorimetric sensor to detect analytes via a color change is feasible for quantitative judgment. After an image is taken, the image analysis results are used in a software program as the standard for quantitative determination to improve detection sensitivity and the objectivity of test results [17,18,19,20,21,22,23,24]. A color immunoassay is thus practical in the field of immunodetection.

The goal of the present research is to develop a new optical rapid test system to upgrade the general rapid test strip detection method from the conventional qualitative analysis to a quantitative analysis. To perform a quantitative analysis, a standard calibration curve with high linearity and low standard deviation at a specific concentration range must be established. In the rapid test system proposed in this study, the image processing program and image detection equipment are combined and tested; the software is programmed using Python, which is beneficial to enhance the reusability of the source code, and the scalability is also excellent, which is potentially beneficial in terms of the integration of the modules. Detection equipment and an image processing program are used to carry out the test. A CMOS sensor is used to capture the image and analyze it to acquire the results of the captured image.

FOB is used as the test target. Currently, only a standard colorimetric card comparison is used as the qualitative judgment. The threshold value for FOB is 50 ng/mL, where if the FOB in the sample exceeds this value, the test result is positive; otherwise, it is considered negative.

Theoretically, the T line should not have any red color if the concentration of the target antigen is below the threshold. A positive result is determined using the naked eye to check if the T line is red in color. This method may cause discrepancies in judgment due to subjective differences in color recognition using the naked eye. If the T line is still slightly red in color before the threshold concentration is reached, the test result may be determined to be positive. Indeed, this is a misjudgment and is thus a false positive. The rapid test system proposed in this paper is aimed toward converting the qualitative detection method into a quantitative detection method. Therefore, the judgments made using the test can be standardized.

## 2. Experimental

### 2.1. System Structure

**Figure 2:**
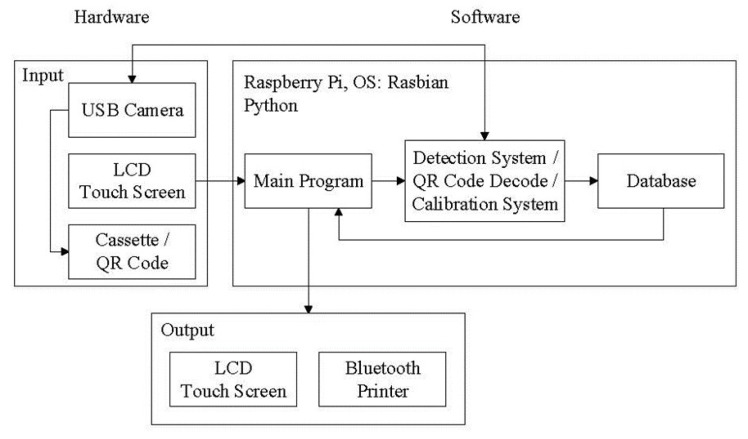
Schematic flow chart of the system signal.

The main process used in this research includes the use of a user interface, image analysis, QR Code decoding, polarization calibration, database storage, and Bluetooth transmission. After capturing a cassette image through a camera and transferring the information to the evaluation unit, the image processing program is then used to analyze the captured image. Finally, the results are shown on the user interface and stored in the database. The system signal flow chart is shown in Figure 2. The main program is a relay station between programs, which serves as a screen display function and a connection point for reading and detection of the results obtained after processing.

The image processing program and the QR Code decoding program are written as independent modules, which are called to execute the designed functions when the main program needs them. Each subprogram is completely closed after execution to release the occupied memory. The detection data from the samples are output to the desired file. This prevents the main program from causing uneven distribution of program memory due to the explosive growth in memory usage when using the camera, which results in call problems between programs.

To use limited memory, an independent module method is used. When one running module is closed, the memory used by this module is released and is used by another ready-to-run module. Thus, only one module is running, which ensures that there will be enough memory to keep the program running.

### 2.2. Image Processing Process

**Figure 3:**
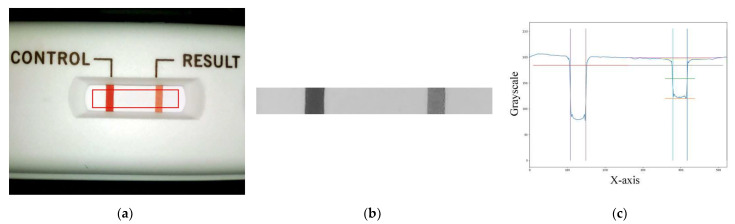
Region-of-interest (ROI) image and grayscale distribution map: (**a**) the captured image of the cassette with the ROI area marked with a red line, (**b**) the captured ROI image, and (**c**) the ROI grayscale distribution map.

The image processing process uses the main program to call the USB camera to take a picture of the cassette (see Figure 3a) in order to obtain a matrix of the 8-bit values of the three primary RGB colors and then to convert the RGB matrix to a grayscale value matrix. The obtained grayscale matrix is used for further region-of-interest (ROI) findings. The ROI area (see Figure 3b) is analyzed for the follow-up analysis. The conversion of the RGB matrix to the grayscale value matrix is applied using Equation (1) as follows:

Gray = 0.299R + 0.587G + 0.114B,(1)

where the R, G, and B components are a number ranging from 0 to 255, respectively. In this study, the R, G, and B components are chosen to be between 125 and 255, where lower numbers mean more red, more green, and more blue, respectively. After the grayscale values are converted, the ROI required for the analysis can be defined. The grayscale value matrix of the ROI can be obtained and averaged to a longitudinal one-dimensional matrix. The plot of the values of the control (C) and T lines along the longitudinal position is shown in Figure 3c.

### 2.3. Hardware Platform

**Figure 4:**
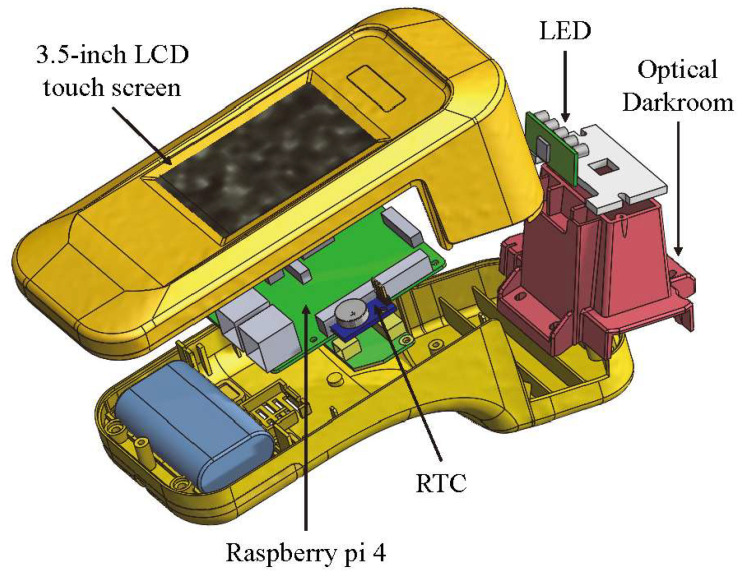
Exploded view of the rapid test system.

The exterior design and internal mechanism layout of the rapid test system were designed by SolidWorks. The system includes a housing, an optical darkroom, and a rapid test cassette carrier. The housing is composed of an upper case and a lower case. The upper case consists of the fixtures for a 3.5-inch LCD touchscreen and a Raspberry Pi 4, while the lower one includes the fixtures for a cooling fan, a real-time clock (RTC) module, and an optical darkroom. The optical darkroom comprises a USB camera, a light guide plate, and an LED circuit board. An exploded view of the rapid test system is shown in Figure 4.

### 2.4. Reagents

According to the experimental requirements, human hemoglobin dry powder (H7378-10G Hemoglobin Human, Sigma-Aldrich, St. Louis, Missouri, USA) was formulated with an FOB extraction buffer (Cat. No. C040-C, Firstep Bioresearch, Inc., Tainan, Taiwan). Then, the different concentrations of FOB specimens used in each experiment were configured into a specimen stock solution. The human hemoglobin solution was shaken with a vortex shaker to avoid any precipitation or unevenness. A micropipette was used to take out the buffer solution, and 1,000,000 ng/mL of FOB stock solution was placed into a centrifuge tube to prepare a 1000 ng/mL FOB solution, which was used as the low-concentration solution. The 1000 ng/mL FOB solution was then mixed with a buffer solution for serial dilution and stored in a refrigerator at 4 °C for use. Before use, it was returned to room temperature and warmed back to 25 °C, after which the experiments were performed. FOB rapid tests (Cat. No. C040-C) were purchased from Firstep Bioresearch, Inc. as well.

## 3. Results and Discussion

**Figure 5:**
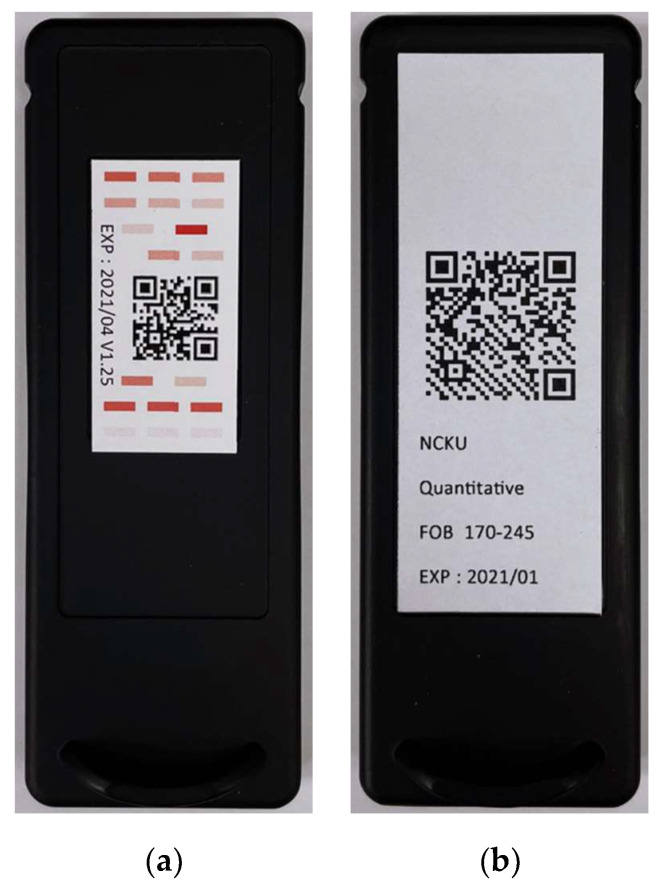
(**a**) The calibration cassette, and (**b**) the QR Code cassette.

Due to slight differences in the LED light intensity and the optical darkroom environments among the rapid test systems, ROI image judgment results may be different for the same tests. Therefore, a calibration cassette was needed to correct the different effects caused by environmental differences among the rapid test systems, thereby reducing deviations in the detection results and thus achieving almost identical detection results among the rapid test systems. The calibration was performed each time the detection system was turned on. The calibration cassette is shown in Figure 5a. The detection system captured the color images on the specific locations of the calibration cassette and calibrated the grayscale values to set numbers. This calibration allowed almost identical results for the same detection systems at different test times or for different detection systems. The QR Code provided the rapid test information input into the rapid test system for further use. The QR Code information contained the calibration curve, the number of sample holes, the test items, and the upper detection limit and lower threshold of the test items. After decoding the QR Code using the rapid test system, the data were read and stored for subsequent use. The QR Code cassette is shown in Figure 5b.

**Figure 6:**
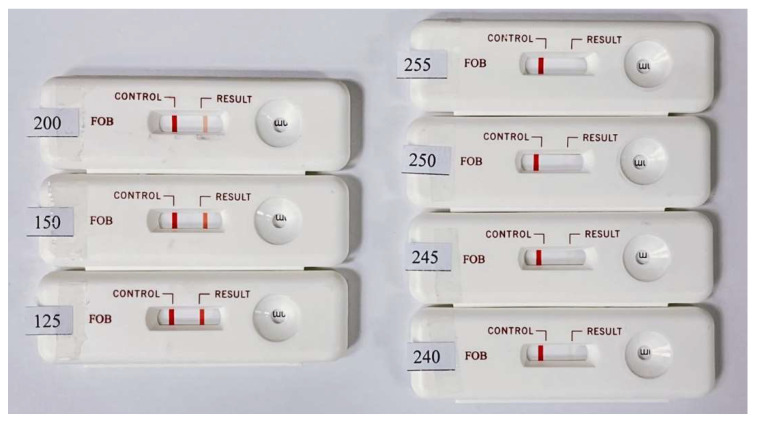
Designed self-made simulated rapid test strips with different grayscale values ranging from 125 to 255 for the T line.

**Figure 7:**
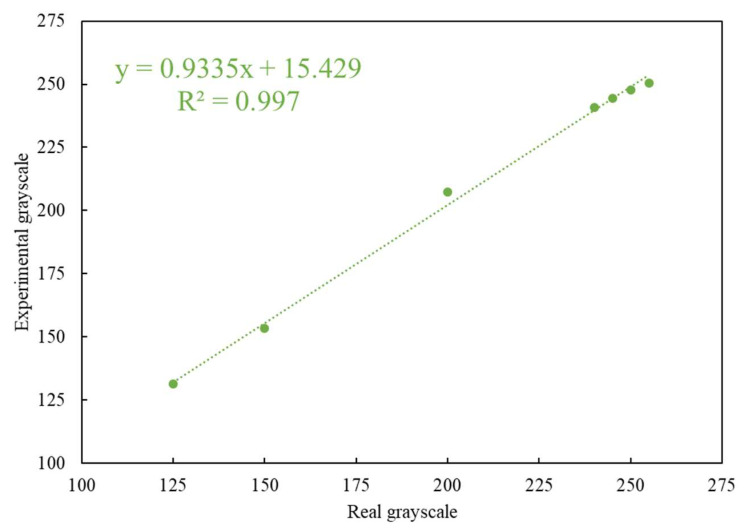
Relationship between the detected experimental grayscales and printed strips with real grayscale.

The colors of the C and T lines for the actual rapid tests were derived from colloidal gold. The coloration changes with the concentration of the colloidal gold nanoparticles in the captured target. Since the color of the colloidal gold ranges between purple and red, self-made simulated color strips close in appearance to the actual test strips were used as the measurement targets. When quantifying the color rendering response through the program analysis, the RGB distribution of an actual immune test strip in the primary colors is close to red. Therefore, the R-values in the three primary RGB colors were fixed, and the values of G and B were changed to design the tests with different color scale values ranging from 125 to 255. These values closely mimic the color changes in real rapid tests. The results obtained by testing the self-made simulated rapid test strips with the rapid test system (see Figure 6) are shown in Figure 7. Figure 7 shows that the detected grayscale values correspond to the printed rapid test strips with real grayscale. It can be seen that the rapid test system has good linearity, and the coefficients of variation (CV) are below 0.4%. The R square value is 0.997. The image analysis program had good linearity for the measurement of different color levels.

**Figure 8:**
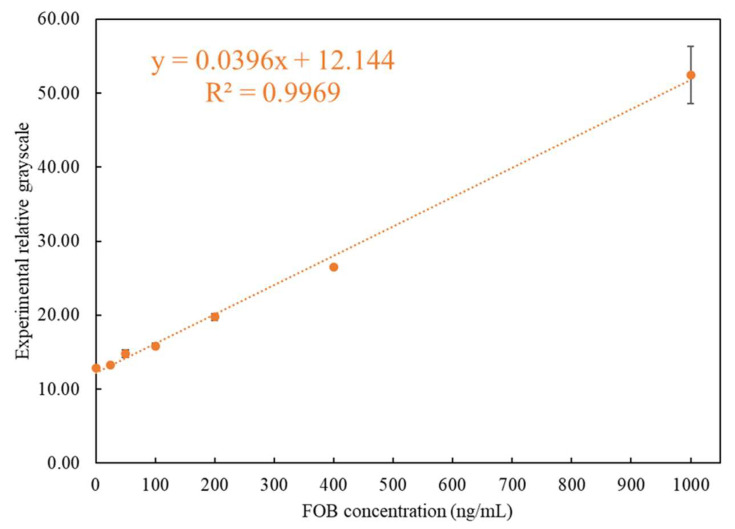
Relationship between the detected experimental grayscales and different concentrations of FOB.

After importing the self-made simulated rapid tests to be tested using the rapid test system, the accuracy of the position, the position range of the C and T lines, and the linearity and sensitivity of the readings were confirmed. Therefore, the experiments were carried out using the rapid test system to perform the tests on actual FOB samples. Seven concentrations of test samples were used for test: 1000 ng/mL, 400 ng/mL, 200 ng/mL, 100 ng/mL, 50 ng/mL, 25 ng/mL, and 0 ng/mL, of which 50 ng/mL was the FOB threshold value. Further, 120 μL of the FOB stool samples with a concentration of 1000 ~ 0 ng/mL was used for the rapid tests. The reaction time was set to five minutes, after which the readout using the rapid test system was performed immediately. After all the required experiments were performed, the average value was taken as the representative value for each concentration. The results of the concentration measured using the rapid test system corresponding to the experimental design concentration are shown in Table 1. When the FOB concentration was below 400 ng/mL, the CV was below 3.7%, and the CV was only 7.41% for 1000 ng/mL. The results are also shown in Figure 8. It can be seen that the analysis of the FOB test strip by the interpretation system had good recognition at a threshold of 50 ng/mL. It was thus verified that the system can provide accurate detection of FOB.

## 4. Conclusions

The use of image interpretation in medical treatment is increasing, and devices using image interpretation technology, such as mobile phones and detection systems, are used as the basis for interpretation to obtain better and more accurate results than have been possible in the past [25]. In this paper, a rapid-test system for FOB detection was successfully developed to provide an immediate quantitative analysis. This made it possible to avoid the instability of the detection results characteristic of general mobile devices that may be caused by ambient light sources and the error results caused by subjective human observation. The proposed rapid-test system was proven to have high sensitivity, linearity, and stability.

Through experimental verification, the feasibility of using image interpretation as a basis for reliable detection was verified. It is hoped that this system can be practically applied in the medical clinical field in the future. A rapid test can quickly and accurately provide initial test results that will help prevent diseases and guide appropriate treatment in the future.

## Figures and Tables

**Table 1:** Relationship between the detected relative experimental grayscale and FOB concentrations.

FOB Concentration (ng/mL)	Relative Grayscale			Average	Standard Deviation	CV
	1st	2nd	3rd			
1000	56.31	52.45	48.53	52.43	3.89	7.41%
400	26.38	26.71	26.43	26.51	0.18	0.67%
200	20.13	19.24	19.85	19.74	0.46	2.30%
100	16.16	15.91	15.42	15.83	0.38	2.37%
50	15.21	14.98	14.18	14.79	0.54	3.65%
25	13.24	13.19	13.31	13.25	0.06	0.45%
0	12.73	12.91	12.79	12.81	0.09	0.71%

## Data Availability

Not applicable.
